# Application of HPLC Fingerprint Coupled with Chemical Pattern Recognition and Multicomponent Quantification for Quality Evaluation of Ethyl Acetate Extract of Banana Leaves

**DOI:** 10.3390/molecules31173023

**Published:** 2026-08-28

**Authors:** Wen Liu, Liyuan Huang, Meifen Ban, Fan Liu, Ze’en Xiao, Xiaotao Hou

**Affiliations:** 1 Faculty of Pharmacy, Guangxi University of Chinese Medicine, Nanning 530200, China; wen_l0639@126.com (W.L.); huang6882024@163.com (L.H.); 13481086337@163.com (M.B.); liufan775116747@gmail.com (F.L.); 2 Key Laboratory of TCM Extraction and Purification and Quality Analysis, Guangxi University of Chinese Medicine, Education Department of Guangxi Zhuang Autonomous Region, Nanning 530200, China; 3 National Demonstration Center for Experimental Traditional Chinese Pharmacology, Guangxi University of Chinese Medicine, Nanning 530200, China; 4 University Engineering Research Center of Reutilization of Traditional Chinese Medicine Resources, Guangxi University of Chinese Medicine, Nanning 530200, China

**Keywords:** ethyl acetate extracts of banana leaves, HPLC, fingerprint, chemical pattern recognition, quality evaluation

## Abstract

Large amounts of banana leaves are discarded as agricultural waste during banana harvesting; however, they are rich in bioactive compounds with promising exploitation potential. The ethyl acetate fraction contains the highest levels of total phenolics and total flavonoids together with strong antioxidant activity, which renders it a candidate source of natural antioxidants. In our current study, HPLC fingerprints were constructed for ten batches of ethyl acetate extracts from banana leaves (BLEA), and fingerprint similarity evaluation coupled with statistical analysis of common peak areas was carried out. Meanwhile, an HPLC quantitative method was developed to simultaneously determine eight characteristic components. The similarity values between the fingerprints of ten batches and the reference fingerprint ‘R’ ranged from 0.791 to 0.988. Cluster analysis (CA) and orthogonal partial least squares discriminant analysis (OPLS-DA) yielded consistent results, revealing clear separation trends among samples from different batches. In this quantitative method, eight characteristic analytes showed favorable linearity within their respective concentration ranges (r ≥ 0.9997). The average recoveries ranged from 101.6% to 104.3%, with RSD of 0.32% to 0.84%, demonstrating that the quantitative method was accurate and reliable. HPLC fingerprint combined with multi-component content determination offers a scientific basis for the quality evaluation and biomass valorization of banana leaves.

## 1. Introduction

**Figure 1:**
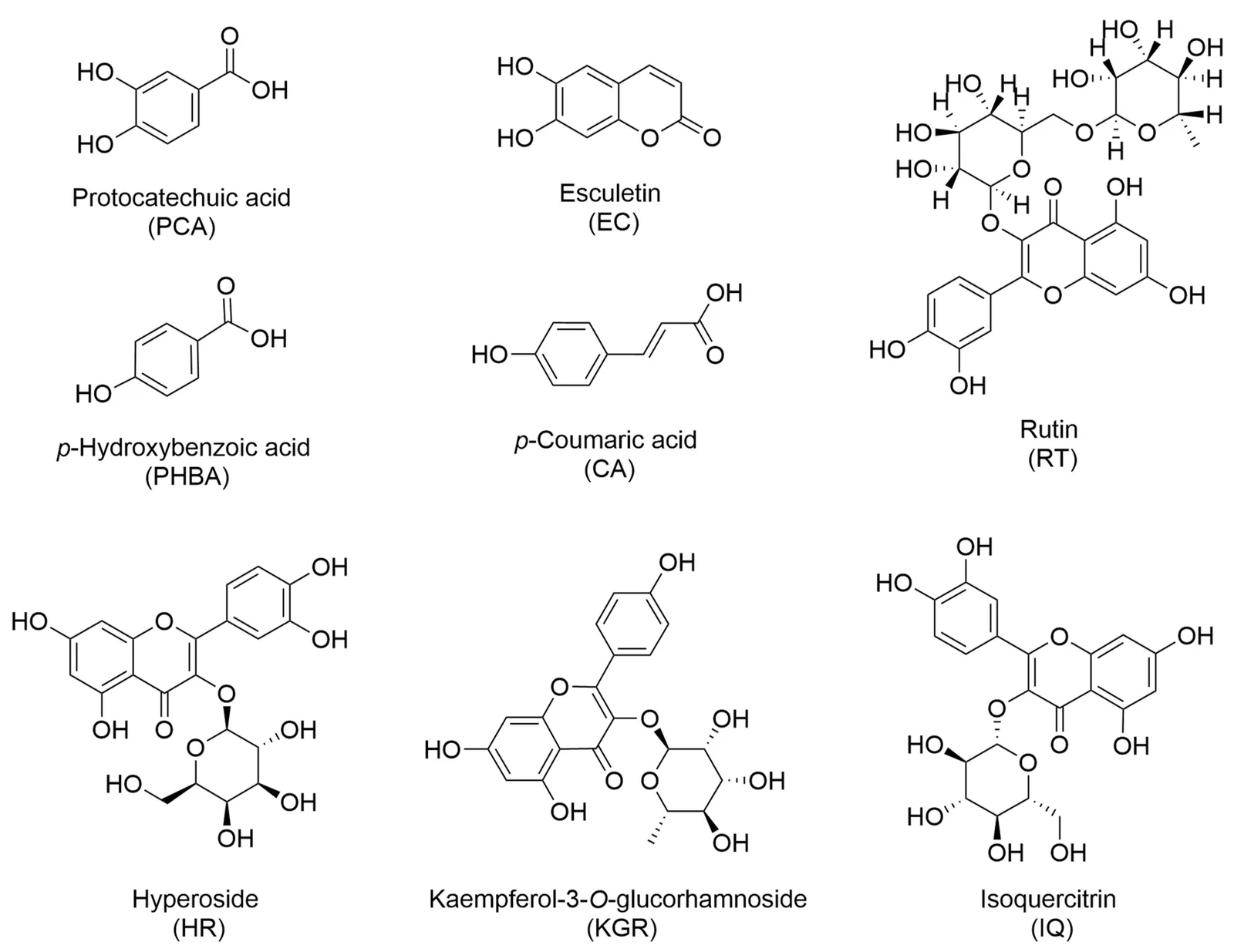
Chemical structure of representative components in BLEA.

*Musa acuminata* Colla (AAA Group), commonly known as banana, is a triploid species belonging to the Musaceae family and plays a significant role in tropical and subtropical agricultural production. The leaves of banana are often discarded as agricultural waste during banana harvesting, with the biomass of these abandoned stems and leaves roughly comparable to the yield of banana fruits. Rational development and efficient utilization of these by-products can enhance the resource utilization rate within the banana industry and mitigate environmental pollution [1,2]. Banana leaves are rich in lignocellulose and exhibit significant potential for resource valorization. Existing studies primarily focus on the green modification of banana leaf fibers [3,4], the preparation of biocomposites [5], the adsorption of water pollutants [6,7], and the biorefinery of lignocellulose [8]. Modern research indicates that banana leaves contain various chemical components, including phenolic acids, flavonoids, terpenes and fatty acids, whose extracts exhibit antioxidant, anti-inflammatory and antibacterial properties [9,10,11,12]. As documented by Widoyanti, A.A.E. [13], banana leaves were extracted with n-hexane, ethyl acetate, ethanol and water under identical detection parameters; the corresponding ethyl acetate extract (BLEA) showed maximum total phenolic and flavonoid contents as well as prominent antioxidant activity. In the preliminary phase of our research, we compared the DPPH and ABTS free radical scavenging capacities of petroleum ether, dichloromethane, ethyl acetate and n-butanol extracts from ten batches of banana leaves. The ethyl acetate fraction exhibited the strongest antioxidant activity, further verifying its outstanding antioxidant potential. This paper focuses on the quality evaluation of BLEA. Variations in geographical environment, cultivation techniques, processing methods and other factors result in certain differences in composition and activity across different batches of banana leaves [14]. The instability of natural drugs’ quality can impact their efficacy, highlighting the necessity for quality control in natural pharmaceuticals. Currently, research on the quality of banana leaves predominantly emphasizes the determination of total phenolics and total flavonoids [15,16,17], leaving specific components inadequately defined. In this study, we established an HPLC fingerprint of BLEA and quantified eight characteristic components, namely protocatechuic acid (PCA), *p*-hydroxybenzoic acid (PHBA), esculetin (EC), *p*-coumaric acid (CA), rutin (RT), hyperoside (HR), kaempferol-3-*O*-glucorhamnoside (KGR) and isoquercitrin (IQ) (Figure 1). Chemical pattern recognition was further applied to assess the quality of this fraction, and this work provides a reference for the rational development and utilization of banana leaf resources. This study introduces an integrated quality evaluation system [18,19,20] that combines fingerprint qualitative analysis, multi-component quantification, and chemical pattern recognition to assess the quality of the ethyl acetate fraction of banana leaves. This approach aims to provide an experimental foundation for the rational development and utilization of waste banana leaf resources.

## 2. Results and Discussion

### 2.1. Method Validation for Fingerprint Analysis

In the precision test, the similarities of the six samples were 0.998, 1.000, 1.000, 1.000, 0.999 and 1.000, respectively. In the stability experiment, the similarities of the seven samples were 1.000, 1.000, 0.998, 1.000, 1.000, 1.000 and 1.000, respectively. In the repeatability experiment, the similarities of the six samples were 1.000, 0.996, 0.996, 1.000, 0.996 and 0.996, respectively. Additionally, the relative retention time and relative peak area of the common peaks were analyzed, revealing that the RSD was less than 3%. These findings indicate that the method exhibits good precision, repeatability and stability, and that the sample solution remains stable at room temperature without any precipitation.

### 2.2. Method Validation for Quantitative Analysis

Mixed standard solutions containing gradient concentrations of PCA, PHBA, EC, CA, RT, HR, KGR and IQ were prepared and analyzed by HPLC. Peak areas were recorded for each injection. Linear regression analysis was conducted, using the mass concentration of reference substances set as the abscissa (X) and the corresponding peak areas as the ordinate (Y). The results are listed in Table 1, which demonstrates that each component exhibited a strong linear relationship within its respective concentration range (r ≥ 0.9997). These results demonstrate the satisfactory performance of the HPLC method and high reliability of the measured data.

The precision of the peak areas was found in the range from 0.29% to 0.77% (see Table S1 for details). The RSD values of retention times were 0.17–0.38% (see Table S2 for details), indicating satisfactory instrument precision. Meanwhile, for the repeatability test, the RSDs were found in the range of 1.2–2.1% (see Table S3 for details), indicating that the method demonstrated good repeatability. The stability test showed that the RSD values of the peak areas for the eight analytes in the test solution over 24 h ranged from 0.37% to 1.8% (see Table S4 for details). The RSD values for retention times were 0.33–0.59% (see Table S5 for details), suggesting that the test solution exhibited good stability within 24 h. Average recoveries of these analytes varied between 101.6 and 104.3% (see Table S6 for details), with RSDs of less than 1%, indicating the good reliability and accuracy of the proposed method.

### 2.3. HPLC Fingerprint Establishment

#### 2.3.1. Identification of Common Peaks

**Figure 2:**
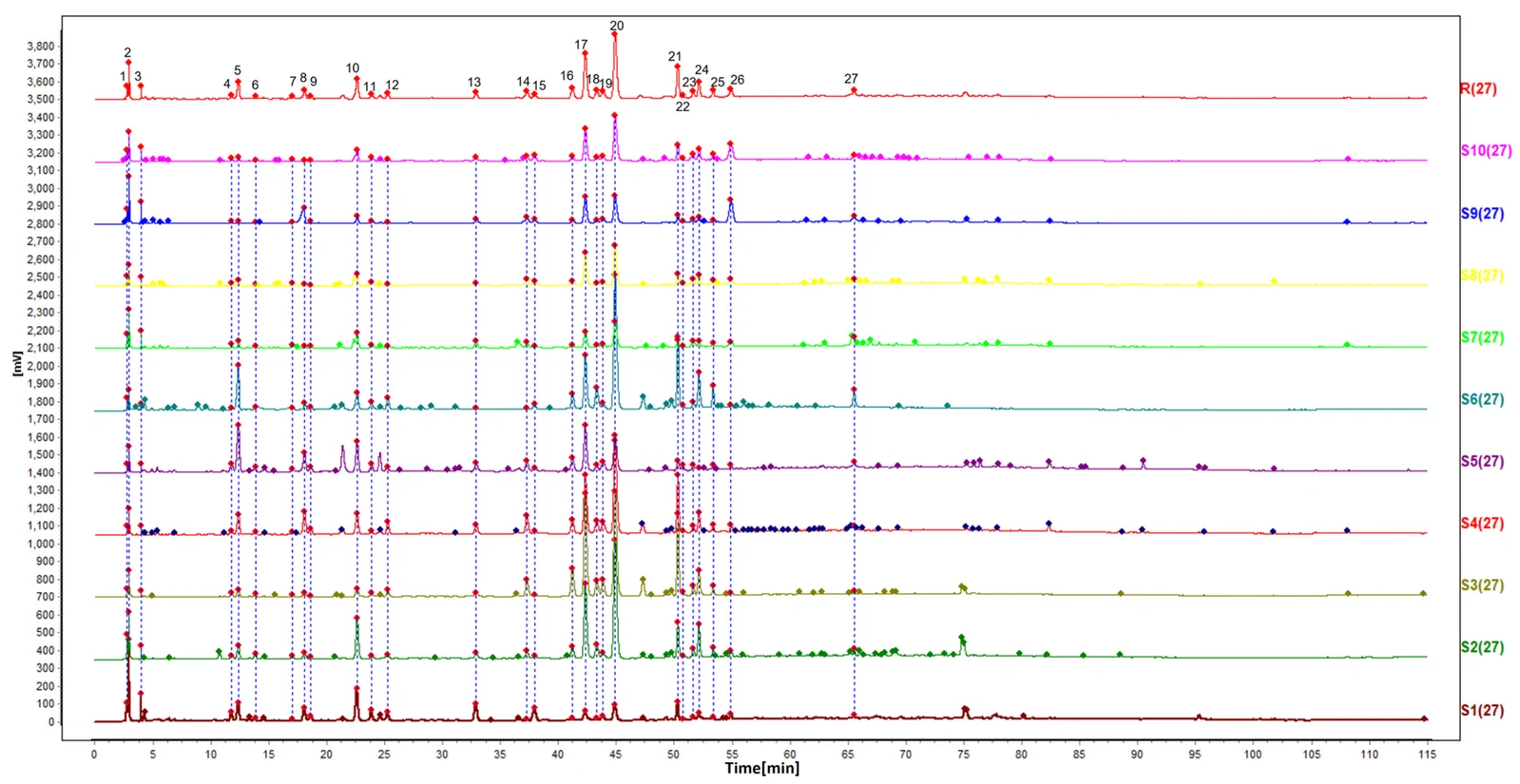
HPLC fingerprints for 10 batches of BLEA. (R, control chromatogram; S1–S10, 10 batches of BLEA fingerprints by HPLC).

Ten batches of BLEA were collected, and the test solution was prepared according to the methodology outlined in Section 3.3.4. The samples were injected into the HPLC system and analyzed under the chromatographic conditions specified in Section 3.3.4. The HPLC fingerprints obtained from the ten batches were imported into the ‘Similarity Evaluation System for Chromatographic Fingerprint of Traditional Chinese Medicine (Version 2012.1)’. S4 was selected as the reference chromatogram, and the median method was applied. Chromatographic peaks were screened with a time window width of 0.5 and a resolution greater than 1.2. Multi-point correction of chromatographic peaks was carried out, followed by peak matching to generate the fingerprints of the ten batches of BLEA. A total of 27 characteristic peaks were labeled with numerical labels (1, 2, 3, …, n) on the overlay chromatogram and standard reference chromatogram (R), as illustrated in Figure 2. The cumulative area of these characteristic peaks accounted for more than 80% of the total peak area, confirming them as common peaks in the HPLC fingerprint of BLEA.

**Figure 3:**
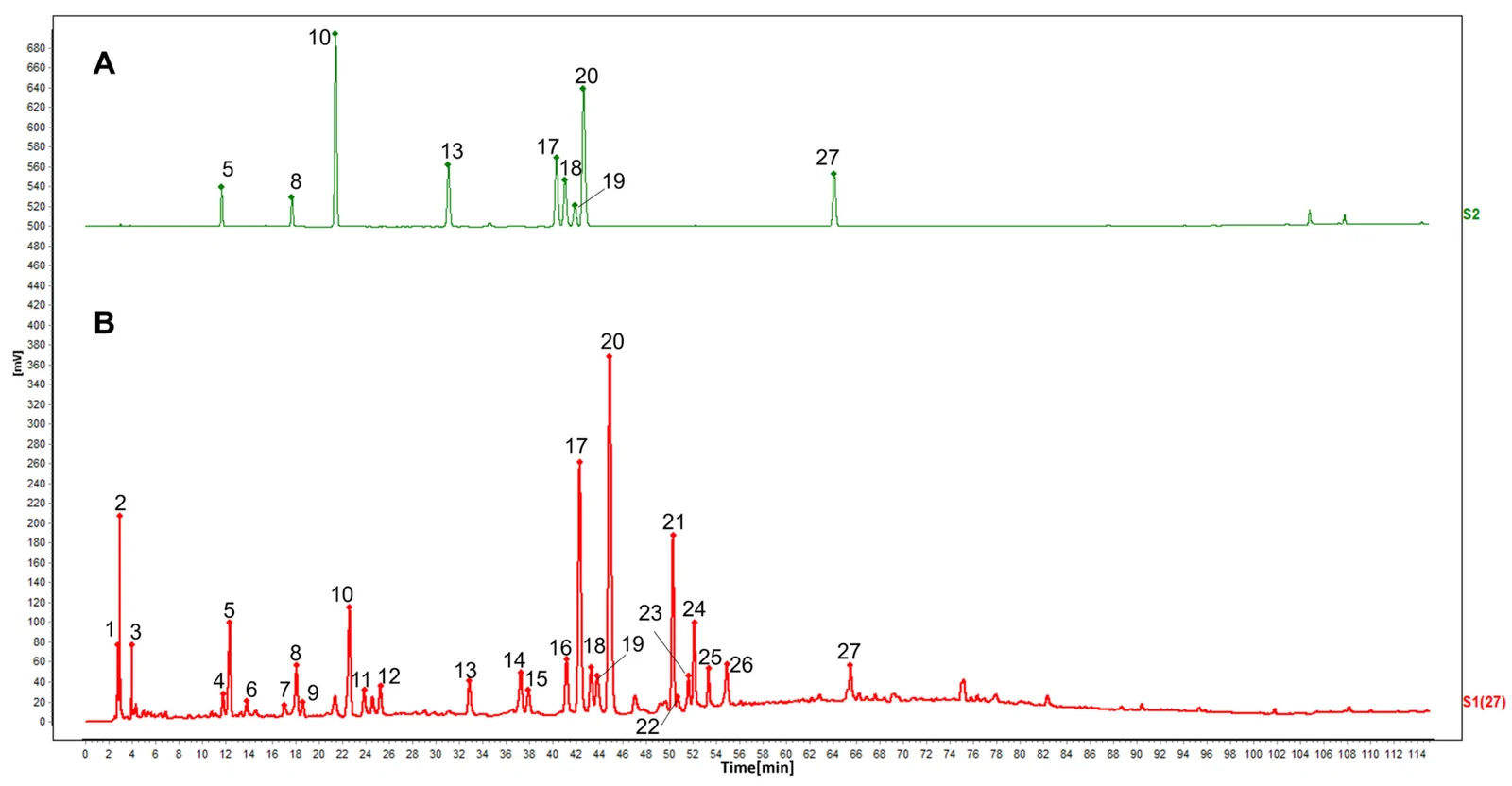
The representative HPLC chromatograms of the standards (**A**) and samples (**B**): 5, PCA; 8, PHBA; 10, EC; 13, CA; 17, RT; 18, KGR; 19, HR; 20, IQ; 27, TCA).

By comparing the retention times of the standards, nine common peaks were identified: PCA (peak 5), PHBA (peak 8), EC (peak 10), CA (peak 13), RT (peak 17), KGR (peak 18), HR (peak 19), IQ (peak 20) and *trans*-cinnamic acid (TCA, peak 27). The control fingerprint (R) of BLEA, along with the superposition diagram of the mixed standard samples, is illustrated in Figure 3.

#### 2.3.2. Similarity Analysis of the HPLC Fingerprint

The similarity values for each batch were calculated by importing chromatograms of BLEA from ten batches and the reference chromatogram into the Similarity Evaluation System for Chromatographic Fingerprint of Traditional Chinese Medicine (Version 2012.1). The similarity results are listed in Table 2. The similarity between each batch of banana leaf samples and reference chromatogram R ranged from 0.791 to 0.988. Most batches exhibited a similarity degree above 0.8 [21], and the majority exceeded 0.9; only one sample yielded a similarity value of 0.791. These findings indicated basically consistent chemical profiles across the ten batches, relatively uniform batch-to-batch quality, and acceptable overall similarity.

### 2.4. Chemical Pattern Recognition

#### 2.4.1. Cluster Analysis (CA)

**Figure 4:**
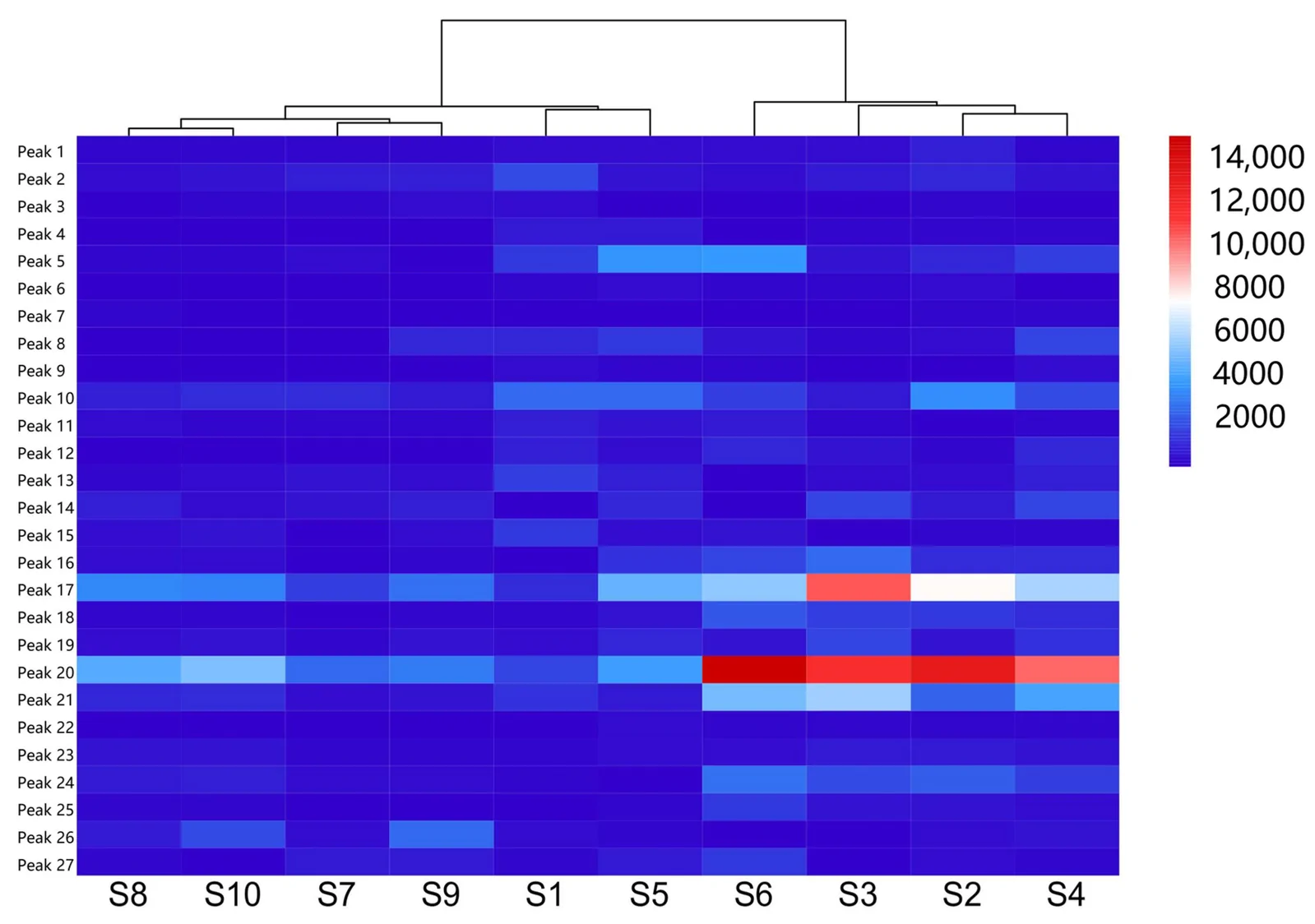
Cluster analysis heat map of 10 batches of BLEA.

The common peak data obtained from chromatographic fingerprints were imported into the online analysis platform (https://hiplot.com.cn/, accessed on 11 May 2026). Ward‘s algorithm was adopted for data processing, and squared Euclidean distance was applied to calculate the chemical similarity of all samples. A cluster heat map was then generated, as shown in Figure 4. The clustering results divided the 10 sample batches into two clusters: Cluster I contained S8, S10, S7, S9, S1 and S5, whereas cluster II consisted of S6, S2, S3 and S4. Such clustering results reveal obvious chemical clustering characteristics and profile differences across different batches of banana leaves.

#### 2.4.2. Orthogonal Partial Least Squares Discriminant Analysis (OPLS-DA)

**Figure 5:**
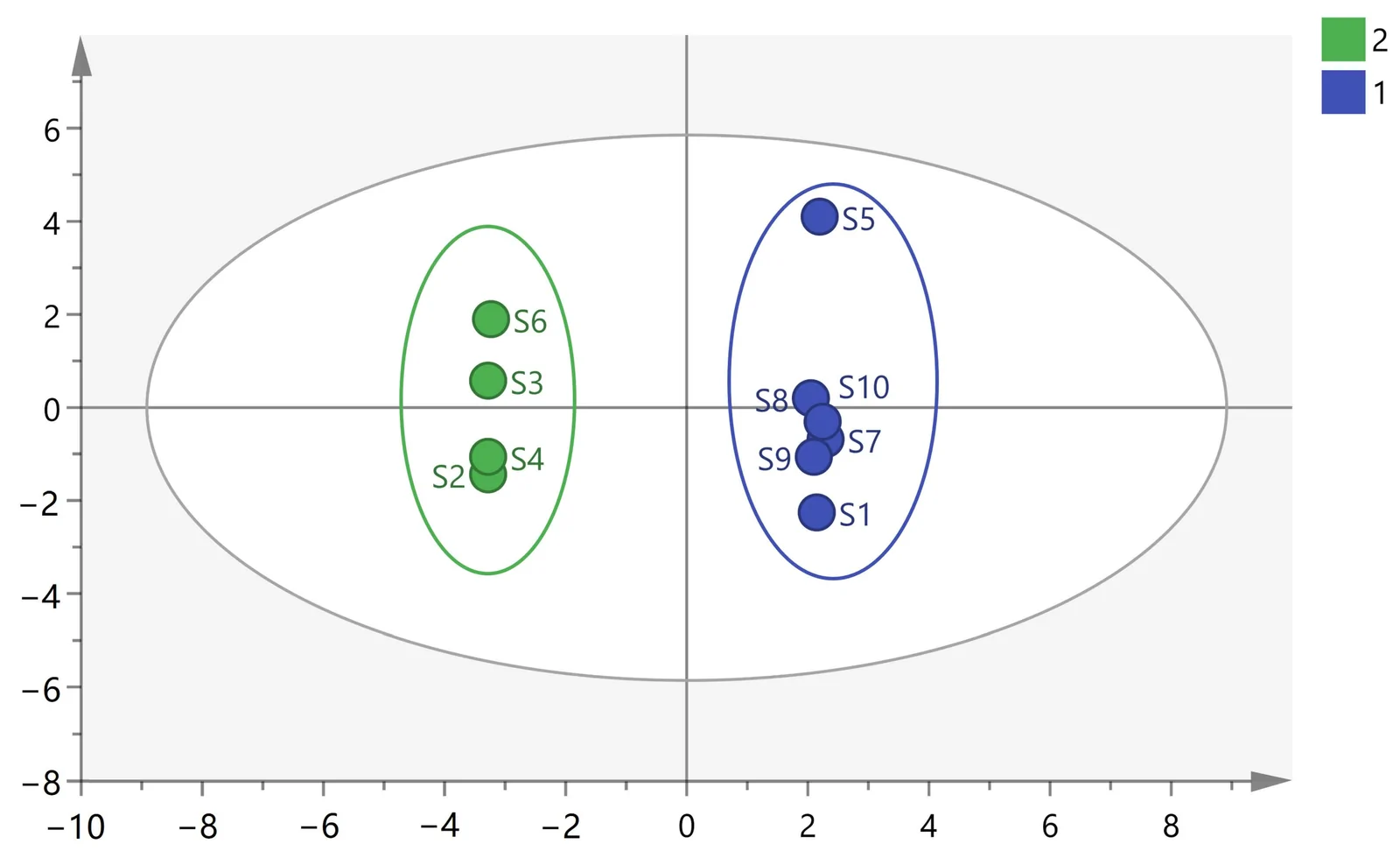
OPLS-DA score plot of 10 sample batches of BLEA.

Common peak area data of ten sample batches were imported into SIMCA 14.1 to construct an OPLS-DA model. The model fitting parameters were obtained as follows: R^2^X = 0.814, R^2^Y = 0.999, and the predictive parameter Q^2^ = 0.92. R^2^X reflects the degree to which the model interprets the X matrix (peak area data), indicating that 81.4% of the original data variation can be explained by the model. R^2^Y reflects the model’s ability to explain the Y matrix (classification information), showing that 99.9% of inter-group differences were captured by the model. Q^2^ is the core index of the model’s predictive ability; its value exceeds 0.5, suggesting that the model demonstrates good predictive ability for the tested samples and meets the evaluation criteria for stability and reliability in chemical pattern recognition. The OPLS-DA score plot of BLEA is presented in Figure 5. The ten sample batches were clustered into two categories, which was consistent with the results of the cluster analysis.

**Figure 6:**
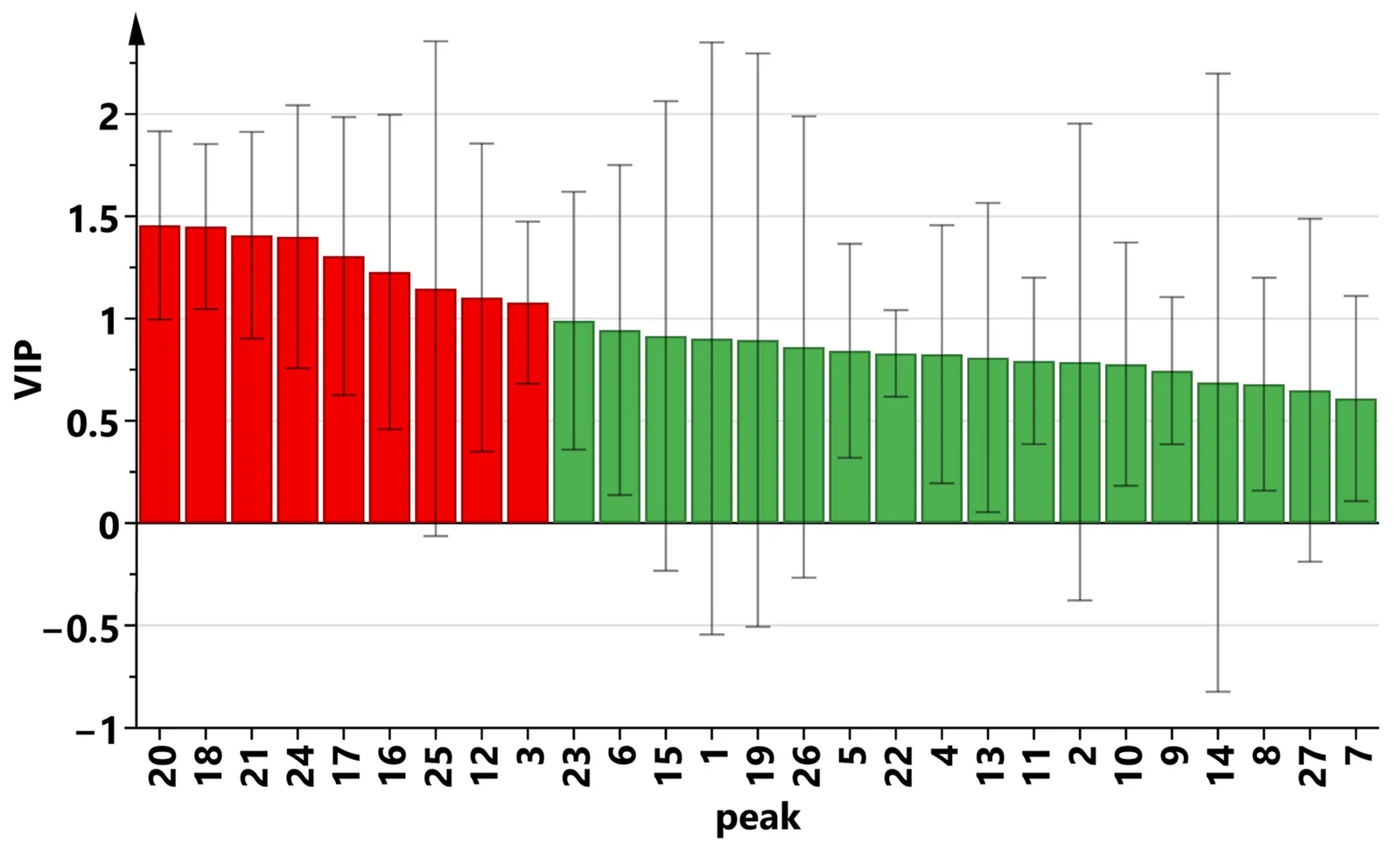
VIP values of 27 chromatographic peaks of 10 batches of BLEA.

The Variable Importance in Projection (VIP) values of the 27 chromatographic peak variables are shown in Figure 6. With a VIP threshold > 1, nine characteristic peaks were screened out: peak 20 (IQ), peak 18 (KGR), peak 21, peak 24, peak 17 (RT), peak 16, peak 25, peak 12 and peak 3. These peaks displayed certain differences among batches of banana leaf samples.

**Figure 7:**
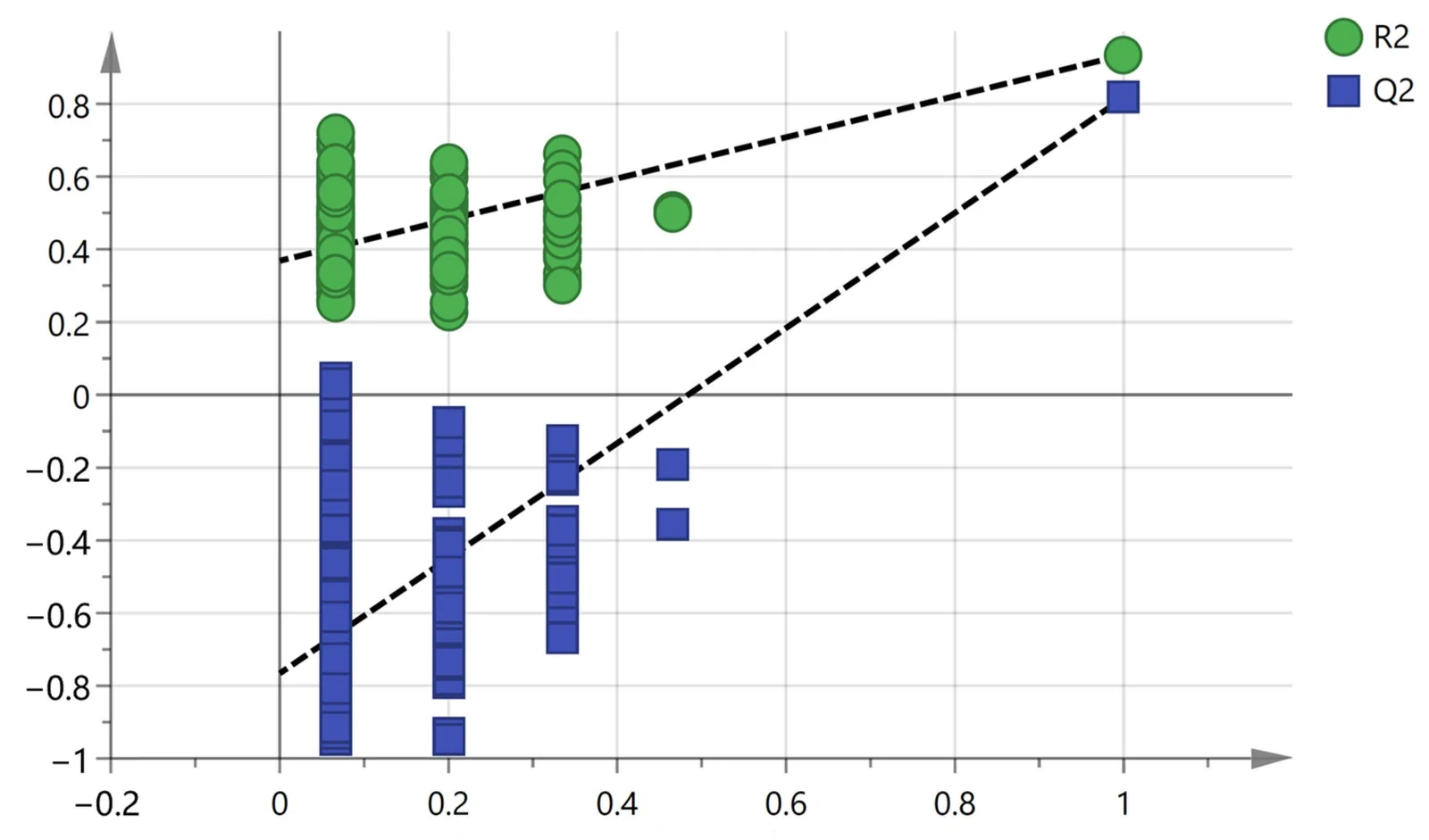
Permutation test of OPLS-DA model for 10 batches of BLEA.

To avoid overfitting of the established OPLS-DA model, which may impair the accuracy of the results, we adopted statistical inference analysis for further model validation, specifically internal validation through 200 permutation tests. The results are presented in Figure 7. The vertical coordinate values of R^2^ and Q^2^ in the upper right corner exceed those on the far left, and the slope is positive. Furthermore, the intersection of the Q^2^ regression line with the vertical axis is less than zero. These findings indicate that the constructed OPLS-DA model is reliable, free from overfitting, and possesses strong predictive capability.

### 2.5. Determination of the Content of Eight Components in BLEA

Among the eight components selected for quantitative analysis, PCA, PHBA and CA are classified as phenolic acids; EC is a coumarin derivative; while RT, HR, KGR and IQ are flavonoid glycosides. All these components exhibit antioxidant and anti-inflammatory activities. They can inhibit the release of pro-inflammatory mediators by regulating signaling pathways such as NF-κB, Nrf2 and NLRP3, thereby exerting anti-inflammatory effects [22,23,24,25,26,27,28,29]. PCA, EC, HR and IQ can mitigate oxidative stress-induced injuries in the heart, liver, kidney and other organs, demonstrating significant organ-protective activities [22,23,26,30]. RT, HR and IQ are capable of preserving vascular endothelial function and alleviating microcirculatory disorders [27,28,30]. KGR can target and modulate respiratory inflammatory responses, exhibiting antitussive and expectorant effects, thus serving as a characteristic active constituent of medicinal plants for the intervention of respiratory inflammation [29]. Additionally, PCA, PHBA, EC and CA demonstrate in vitro bacteriostatic effects and potential anti-tumor pharmacological activities [22,24,25,26]. Therefore, these eight components were selected as the analytes for quantitative determination in this study.

Ten batches of BLEA were taken and prepared into sample solutions according to the method described in Section 3.3.4. After the sample solutions were injected into the HPLC and analyzed under the chromatographic conditions described in Section 3.3.4, peak areas were recorded, and the contents of eight characteristic components (PCA, PHBA, EC, CA, RT, HR, KGR and IQ) were calculated using the regression equations (Table 3). Quantitative results of characteristic components revealed certain differences in the contents of common constituents across different batches of banana leaves. The contents of the eight components varied among different batches of banana leaves, which may be attributed to variations in climatic conditions, soil environment, harvesting time, cultivation management and the growth cycles of the producing areas. IQ showed overwhelmingly higher levels in all six batches, with a maximum concentration reaching 614.9 mg/g. As a flavonol glycoside, IQ possesses multiple pharmacological activities, including antioxidant, anti-inflammatory, cardiovascular-protective and glucose-regulating effects [30,31,32]. Compared to quercetin, IQ demonstrates superior water solubility and oral bioavailability. Consequently, banana leaves present significant advantages for the development of natural antioxidant functional raw materials.

## 3. Materials and Methods

### 3.1. Plant Materials, Chemicals, and Reagents

Different batches of banana leaves were sourced from Guangdong and Guangxi, China. These leaves were identified as belonging to the species *Musa acuminata* Colla (AAA) by Associate Professor Dai Zhonghua from the Department of Pharmaceutical Botany at Guangxi University of Chinese Medicine. The details of the samples are presented in Table 4. The reference substances used in the experiments, including PCA, PHBA, EC, CA, RT, HR, KGR and IQ, were procured from Chengdu Medison Technology Co., Ltd., Chengdu, China, with a purity exceeding 98%. Chromatographic-grade acetonitrile was obtained from Symmerfels Technology (China) Co., Ltd., Shanghai, China. Additionally, analytical methanol, ethanol, petroleum ether, dichloromethane and ethyl acetate were sourced from Chengdu Kelong Chemical Co., Ltd., Chengdu, China, while pure phosphoric acid was acquired from National Pharmaceutical Group Chemical Reagents Co., Ltd., Shanghai, China.

### 3.2. Apparatus

The study utilized an Agilent 1100 HPLC system, which comprises a G1311A four-way pump (Agilent Technologies, Santa Clara, CA, USA), a G1322A degasser, a G1313A automatic sampler, a G1315B diode array detector and a G1316A column oven. System control and data analysis were performed using the M8307AA chromatographic workstation. Additional equipment included an electronic balance (ME204/02, Mettler-Toledo Instruments (Shanghai) Co., Ltd., Shanghai, China), a circulating water multi-purpose vacuum pump (UPC-II-10T, Zhengzhou Great Wall Technology Industry and Trade Co., Ltd., Zhengzhou, China), an ultrasonic cleaner (KQ5200B, Kunshan Ultrasonic Instrument Co., Ltd., Kunshan, China), and a pure water meter (UPC-II-10T, Sichuan Youpu Ultra-pure Technology Co., Ltd., Chengdu, China).

### 3.3. Fingerprint of BLEA

#### 3.3.1. Screening of Solvents for Sample Dissolution

The effects of 50% methanol, 70% methanol and 100% methanol on chromatogram performance were systematically investigated. Key chromatographic parameters, including peak type, peak area and resolution, were comprehensively evaluated. Ultimately, 70% methanol was determined to be the optimal solvent for sample dissolution.

#### 3.3.2. Screening of Fingerprint Detection Wavelengths

From the perspective of information theory, each chromatographic peak in fingerprints can be regarded as an independent discrete information unit, where the peak area reflects the abundance of corresponding chemical constituents. The information entropy (*H*) calculated from peak area data can quantitatively characterize the distribution probability of chemical components and further evaluate the information richness of chromatograms under different detection conditions [33]. DAD full-wavelength scanning of BLEA sample solutions was performed over the range of 200–400 nm with data collected at 2 nm intervals. Information entropy values were calculated according to the reported calculation procedure [34], and the corresponding formula is shown below:

$$H=-\frac{1}{\ln2}\sum_{i=1}^{n}Wi\ln Wi$$

The maximum information entropy value corresponded to the chromatogram with the most abundant peak information and the largest number of detectable peaks. After excluding interference from terminal absorption and background noise, 226 nm, the wavelength yielding the highest entropy (*H*) (Figure S1), was determined to be the optimal detection wavelength for fingerprint analysis, which afforded a balanced high signal-to-noise ratio and complete component peak information.

#### 3.3.3. Examination of Other Chromatographic Conditions

Four mobile phase combinations were further screened in this work: acetonitrile–0.1% aqueous phosphoric acid, acetonitrile–0.1% aqueous formic acid, acetonitrile–0.1% aqueous acetic acid and acetonitrile–pure water (Figures S2–S5). Meanwhile, three column temperatures (25 °C, 30 °C, 35 °C) were investigated (Figures S6–S8). The finalized chromatographic gradient elution conditions for fingerprint profiling were set as follows: mobile phase A, acetonitrile; mobile phase B, 0.1% aqueous phosphoric acid; flow rate, 1.0 mL/min; injection volume, 20 μL; column temperature, 35 °C; detection wavelength, 226 nm.

#### 3.3.4. Determination of Fingerprints

Approximately 50 g of banana leaf powder (sieved through a 20 mesh) was accurately weighed after being dried in the shade. The powder was refluxed and extracted twice with 500 mL of 70% ethanol for 1 h each time. The extracts were combined, filtered, and concentrated under reduced pressure to a final volume of 200 mL. Subsequently, petroleum ether, dichloromethane and ethyl acetate were added sequentially, following their polarity from low to high, until the organic phase became approximately transparent and colorless. After concentrating the solvent under reduced pressure, the extract was transferred to an evaporator and evaporated to dryness in a water bath, yielding the BLEA. Approximately 50 mg of BLEA was accurately weighed, and 70% methanol was added to dissolve it, bringing the total volume to 10 mL. After filtration through a 0.45 μm microporous membrane, the test solution was obtained. Appropriate amounts of nine reference standards were accurately weighed and dissolved in 70% methanol to prepare a mixed standard solution. The concentrations of the analytes in the mixed solution were as follows: PCA 22.30 µg/mL, PHBA 30.60 µg/mL, EC 142.1 µg/mL, CA 66.40 µg/mL, RT 93.20 µg/mL, KGR 48.10 µg/mL, HR 105.0 µg/mL, IQ 112.1 µg/mL and TCA 86.40 µg/mL. The separation was conducted using an Elite Supersil ODS2 column (4.6 mm × 250 mm, 5 μm). The mobile phase comprised acetonitrile and a 0.1% phosphoric acid aqueous solution.

An injection volume of 20 μL was utilized and the column was maintained at a temperature of 35 °C. The detection wavelength was set at 226 nm. The gradient elution conditions are detailed in Table S7.

### 3.4. Verification of HPLC Fingerprint Method

The BLEA of S4 sample was accurately weighed, and the test solution was prepared according to the method outlined in Section 3.3.4. Subsequently, the solution was injected continuously six times under the chromatographic conditions specified in Section 3.3.4. The relative peak areas and relative retention times of each main chromatographic peak were recorded. Using peak 17 (RT) as the reference peak, the relative retention time relative standard deviation (RSD) and relative peak area RSD for each common peak were calculated to assess the instrument’s precision.

The BLEA of S4 was accurately weighed, and the test solution was prepared according to the method described in Section 3.3.4. Subsequently, the sample was injected and analyzed at 0 h, 2 h, 4 h, 8 h, 10 h, 12 h, and 24 h, following the chromatographic conditions outlined in Section 3.3.4. Peak 17 (RT) was selected as the reference peak, and the relative retention time RSD and relative peak area RSD for each common peak were calculated to assess the stability of the test solution.

The BLEA of S4 was accurately weighed, and the test solution was prepared following the method outlined in Section 3.3.4, with six parallel samples. Subsequently, continuous injections were performed according to the chromatographic conditions specified in Section 3.3.4. Using peak 17 (RT) as the reference peak, the relative retention time RSD and relative peak area RSD for each common peak were calculated to assess the method’s repeatability.

### 3.5. Content Determination of Eight Components in BLEA

#### 3.5.1. Preparation of Mixed Reference Solution and Sample Solution

Accurately weighed reference standards were dissolved in 70% methanol to prepare a mixed standard solution. The concentrations of the analytes were as follows: PCA 0.2230 mg/mL, PHBA 0.3060 mg/mL, EC 1.421 mg/mL, CA 0.6640 mg/mL, RT 0.9320 mg/mL, KGR 0.4810 mg/mL, HR 1.050 mg/mL and IQ 1.121 mg/mL. The prepared solution was stored at 4 °C in a refrigerator.

The preparation method for the sample solution follows the procedure outlined in Section 3.3.4.

#### 3.5.2. Chromatographic Conditions

Eight characteristic constituents were identified from the common fingerprint peaks (PCA, PHBA, EC, CA, RT, HR, KGR and IQ). DAD full-wavelength scanning showed that all eight target compounds displayed strong UV absorption and satisfactory chromatographic separation at 264 nm. Accordingly, 264 nm was chosen as the detection wavelength for subsequent quantitative determination of these components. The detection wavelength for the chromatographic conditions described in Section 3.3.4 has been adjusted from 226 nm to 264 nm, while all other conditions remain unchanged.

#### 3.5.3. Validation of the Quantitative Method

Accurate measurements of mixed control solutions (0.5 mL, 0.8 mL, 1.0 mL, 1.2 mL and 1.5 mL) were performed and placed into a 10 mL volumetric flask. Subsequently, 70% methanol was added to achieve the desired volume. Each solution was then injected into the HPLC. The analysis followed the methodology outlined in Section 3.5.2, and the resulting chromatograms were recorded. A calibration curve was constructed using the mass concentration of the reference substance on the x-axis and the corresponding chromatographic peak area on the y-axis.

The accuracy was assessed through the continuous injection of the same batch of banana leaf sample solution (S4) for six repetitions. Stability tests were conducted using the same sample solution at intervals of 0, 2, 4, 8, 10, 12 and 24 h. Additionally, six replicates of the same batch of banana leaf sample solution (S4) were prepared in parallel to evaluate repeatability. The relative standard deviation (RSD) of the peak area and retention time for PCA, PHBA, EC, CA, RT, HR, KGR and IQ were employed as the determination indices.

The sample recovery test was conducted to verify the accuracy of the method. Approximately 25 mg of S4 batch samples were accurately weighed. A reference substance was added according to 100% of the content of the components to be tested in the sample. Subsequently, 70% methanol was added to dissolve the mixture, and the volume was adjusted to 10 mL, with six parallel preparations made. The average recovery rate was evaluated by calculating the ratio of the detected amount to the added amount, in accordance with the conditions outlined in Section 3.5.2.

### 3.6. Data Analysis

Peak calibration and similarity analysis were conducted in accordance with the ‘Similarity Evaluation System for Chromatographic Fingerprint of Traditional Chinese Medicine’ (v. 2012) as recommended by the Chinese Pharmacopoeia Commission. The similarity was calculated using the vector angle cosine method. Additionally, SIMCA 14.1 software was employed to perform orthogonal partial least squares discriminant analysis (OPLS-DA) on the chromatographic peak areas of 27 components found in BLEA.

## 4. Conclusions

As an agricultural waste, banana leaves have garnered significant research interest from numerous scholars. To date, researchers have isolated various active substances from banana plants. These active components confer notable pharmacological functions to bananas, including antioxidant, immunomodulatory, antibacterial, lipid-lowering, and hypoglycemic activities. Bananas exhibit promising potential for enhancing human health and preventing diseases. However, studies focusing on the quality control of banana leaves remain scarce. This paper investigates the HPLC fingerprints of banana leaves and establishes a quantitative method for eight active components, thereby laying an experimental foundation for the development of the medicinal value of banana leaves. In this study, the quality of banana leaf samples was evaluated using HPLC fingerprinting, combined with chemical pattern recognition and multi-component content determination. The HPLC fingerprint of ethyl acetate extracts from banana leaves was successfully established. The similarity between the fingerprints of 10 batches of samples and the control fingerprint R ranged from 0.791 to 0.988, which revealed certain differences in the chemical composition of banana leaf ethyl acetate extracts across different batches. Cluster analysis and orthogonal partial least squares discriminant analysis were employed to analyze the 27 characteristic peaks. Notably, nine common chemical components—peak 20 (isoquercitrin), peak 18 (kaempferol-3-*O*-glucorhamnoside), peak 21, peak 24, peak 17 (rutin), peak 16, peak 25, peak 12, and peak 3 showed certain differences across the various batches of banana leaves. Six of these peaks remain unidentified, necessitating further research for their identification. The detection results indicated that the contents of eight components varied among different batches of banana leaves, likely due to differences in origin, harvesting date and leaf maturity. Although the samples in this study were collected from diverse sources with stable detection data, supporting preliminary methodological exploration, the sample size was limited. Consequently, the specific causes of the quality differences require further investigation in subsequent research. Future research will expand the sample size by including various producing areas, harvesting periods, cultivation conditions and growth cycles. This approach aims to further investigate the intrinsic causes of sample quality variation and to identify high-quality raw materials with elevated levels of active constituents. Such findings will provide scientific evidence to support the development and utilization of banana leaves in the health sector.

## Figures and Tables

**Table 1:** Results of the validation procedure for reference substances.

Analytes	Regression Equation	R	Linear Range (µg/mL)	Precision (RSD%, *n* = 6)	Repeatability (RSD%, *n* = 6)	Stability (RSD%, *n* = 7)	Recovery (%, *n* = 6)	
							Average	RSD%
PCA	y = 29,732.4x + 5.13593	0.9998	11.15~33.45	0.33	1.2	0.98	102.2	0.32
PHBA	y = 47,136.2x + 12.6960	0.9997	15.30~45.90	0.37	1.8	0.47	103.6	0.37
EC	y = 9981.96x + 15.1453	0.9997	71.05~213.2	0.30	1.3	0.63	103.7	0.84
CA	y = 19,790.7x + 12.3335	0.9997	33.20~99.60	0.38	1.8	1.7	102.0	0.45
RT	y = 16,795.4x + 12.7298	0.9997	46.60~139.8	0.55	1.9	0.37	101.6	0.66
HR	y = 23,260.5x + 16.3820	0.9998	52.50~157.5	0.43	2.2	0.68	104.3	0.52
KGR	y = 18,638.4x + 7.01948	0.9997	24.05~72.15	0.29	2.1	1.8	101.9	0.60
IQ	y = 23,636.0x + 23.7638	0.9997	56.05~168.2	0.77	2.0	1.1	103.9	0.58

Notes: y, Peak area; x, the concentration of standards (µg/mL).

**Table 2:** Similarity of fingerprints of 10 batches of BLEA.

	S1	S2	S3	S4	S5	S6	S7	S8	S9	S10	Reference Fingerprint R
S1	1.000	0.722	0.638	0.759	0.872	0.700	0.873	0.736	0.738	0.738	0.791
S2	0.722	1.000	0.939	0.967	0.818	0.951	0.936	0.975	0.822	0.958	0.982
S3	0.638	0.939	1.000	0.959	0.792	0.906	0.859	0.956	0.802	0.915	0.958
S4	0.759	0.967	0.959	1.000	0.842	0.959	0.916	0.962	0.835	0.937	0.988
S5	0.872	0.818	0.792	0.842	1.000	0.782	0.854	0.839	0.769	0.793	0.873
S6	0.700	0.951	0.906	0.959	0.782	1.000	0.877	0.915	0.747	0.901	0.957
S7	0.873	0.936	0.859	0.916	0.854	0.877	1.000	0.949	0.89	0.938	0.951
S8	0.736	0.975	0.956	0.962	0.839	0.915	0.949	1.000	0.889	0.979	0.984
S9	0.738	0.822	0.802	0.835	0.769	0.747	0.89	0.889	1.000	0.925	0.867
S10	0.738	0.958	0.915	0.937	0.793	0.901	0.938	0.979	0.925	1.000	0.965
Reference Fingerprint R	0.791	0.982	0.958	0.988	0.873	0.957	0.951	0.984	0.867	0.965	1.000

Notes: S1–S10, Sample 1–10.

**Table 3:** Contents of eight characteristic components in 10 batches of BLEA.

NO	Content (mg/g)							
	PCA	PHBA	EC	CA	RT	HR	KGR	IQ
S1	49.40	19.92	261.7	82.19	65.33	9.699	25.40	72.49
S2	34.30	9.271	340.1	22.73	435.3	60.19	30.25	530.9
S3	18.23	6.072	69.64	18.38	611.5	70.42	91.14	475.0
S4	53.40	37.49	184.6	46.03	341.5	51.78	69.75	419.4
S5	122.1	31.43	262.8	44.72	271.0	21.42	55.41	165.1
S6	126.9	4.152	153.0	8.919	316.1	91.15	32.68	614.9
S7	13.03	2.112	107.0	29.96	94.40	7.335	16.60	111.7
S8	10.23	2.065	89.00	14.57	194.0	9.030	20.06	180.0
S9	3.691	21.32	69.18	22.26	158.5	9.212	32.56	129.5
S10	7.337	1.288	113.0	19.57	191.4	13.76	26.95	211.5

Notes: S1–S10, Sample 1–10.

**Table 4:** Details of banana leaf samples from different habitats.

No	Habitat	Collection Date
S1	Wuming District, Nanning City, Guangxi Province, China	16 September 2024
S2	Machong Town, Dongguan City, Guangdong Province, China	3 January 2025
S3	Qingxiu District, Nanning City, Guangxi Province, China	26 October 2024
S4	Xixiangtang District, Nanning City, Guangxi Province, China	16 September 2024
S5	Shuangqiao Town, Nanning City, Guangxi Province, China	10 September 2024
S6	Liangqing District, Nanning City, Guangxi Province, China	16 September 2024
S7	Qinzhou Qinnan District, Guangxi Province, China	18 September 2024
S8	Tanluo Town, Nanning City, Guangxi Province, China	16 November 2025
S9	Yuancheng District, Heyuan City, Guangdong Province, China	20 November 2025
S10	Xixiangtang District, Nanning City, Guangxi Province, China	22 October 2025

Notes: S1–S10, Sample 1–10.

## Data Availability

The original contributions presented in this study are included in the article/Supplementary Material. Further inquiries can be directed to the corresponding authors.

## Supplementary Materials

The following supporting information can be downloaded at https://www.mdpi.com/article/10.3390/molecules31173023/s1. Table S1: The results of peak area precision test of each component (*n* = 6); Table S2: The results of retention time precision test of each component (*n* = 6); Table S3: Repeatability test results (*n* = 6); Table S4: The results of peak area stability test of each component (*n* = 6); Table S5: The results of retention time stability test of each component (*n* = 6); Table S6: Sample recovery test results (*n* = 6); Table S7: The gradient elution conditions; Figure S1: Curves of information entropy under different detection wavelengths; Figure S2: Chromatogram with acetonitrile–0.1% phosphoric acid as mobile phase (226 nm); Figure S3: Chromatogram with acetonitrile–0.1% formic acid as mobile phase (226 nm); Figure S4: Chromatogram with acetonitrile–0.1% acetic acid as mobile phase (226 nm); Figure S5: Chromatogram with acetonitrile–water as mobile phase (226 nm); Figure S6: Chromatogram at column temperature of 25 °C; Figure S7: Chromatogram at column temperature of 30 °C; Figure S8: Chromatogram at column temperature of 35 °C; Figure S9: Chromatogram of S4 detected at 272 nm (red) and 264 nm (blue).

## Abbreviations

The following abbreviations are used in this manuscript:

BLEA	Ethyl acetate extracts from banana leaves
HPLC	High performance liquid chromatography
CA	Cluster analysis
OPLS-DA	Orthogonal partial least squares discriminant analysis
RSD	Relative standard deviation
PCA	Protocatechuic acid
PHBA	*p*-Hydroxybenzoic acid
EC	Esculetin
CA	*p*-Coumaric acid
RT	Rutin
HR	Hyperoside
KGR	Kaempferol-3-*O*-glucorhamnoside
IQ	Isoquercitrin
TCA	*trans*-Cinnamic acid

## References

[1] Zou F., Tan C., Zhang B., Wu W., Shang N. (2022). The valorization of banana by-products: Nutritional composition, bioactivities, applications, and future development. Foods.

[2] Gayathry K.S., John J.A. (2015). Banana by-products as an emerging and sustainable source of bioactive compounds-insights and applications. Biomass Convers. Biorefin..

[3] Marinho E., Chaves D.M., Araújo J.C., Michelin M., Fangueiro R., Ferreira D.P. (2026). Exploring fiber characteristics: Comparative analysis of mechanically extracted banana leaf central rib fibers from Azores and Madeira. Chem. Eng. J. Green Sustain..

[4] Ngo H.T., Pham M.T., Dao Y.H. (2026). Fully green and scalable solvent-free functionalization of banana waste fibers for high-performance biodegradable thermoplastic starch/DES composites. Compos. Interfaces.

[5] Tarrés Q., Espinosa E., Domínguez-Robles J., Rodríguez A., Mutjé P., Delgado-Aguilar M. (2017). The suitability of banana leaf residue as raw material for the production of high lignin content micro/nano fibers: From residue to value-added products. Ind. Crops Prod..

[6] Behera B., Arti, Mehra R. (2025). Removal of uranium from aqueous solution using cellulose extracted from the leaves of *Musa paradisiaca*. Appl. Radiat. Isot..

[7] Rodrigues J., Silva M., Pinto F. (2025). Mechanisms of phenol adsorption on banana leaves and coffee husk biochars. ACS Omega.

[8] Yu X., Li Y., Xu S. (2024). Optimization of extraction process of banana pseudostem fibers and its characterization. Biomass Chem. Eng..

[9] Sánchez-Rodríguez L., Hernández M., Martín J., Pérez A., Rodríguez C., Fernández M., González P. (2025). Valorization of banana leaf (*Musa* spp. var. Cavendish): A comprehensive study of bioactive compounds and biological activities. Biocatal. Agric. Biotechnol..

[10] Sonibare M.A., Ayoola I.O., Gueye B., Abberton M.T., D’Souza R., Kuhnert N. (2018). Leaves metabolomic profiling of *Musa acuminata* accessions using UPLC-QTOF-MS/MS and their antioxidant activity. J. Food Meas. Charact..

[11] Yudhit A., Pintauli S., Herda E., Dalimunthe A. (2026). The antibacterial activity of Barangan (*Musa acuminata* Colla) peel on *Streptococcus mutans*: In silico and in vitro study. Dent. Mater. J..

[12] Mostafa H.S. (2021). Banana plant as a source of valuable antimicrobial compounds and its current applications in the food sector. J. Food Sci..

[13] Widoyanti A.A.E., Chaikong K., Rangsinth P., Saengratwatchara P., Leung G.P.H., Prasansuklab A. (2023). Valorization of Nam Wah banana (*Musa paradisiaca* L.) byproducts as a source of bioactive compounds with antioxidant and anti-inflammatory properties: In vitro and in silico studies. Foods.

[14] Ayoola-Oresanya I.O., Sonibare M.A., Gueye B., Paliwal R., Abberton M.T., Morlock G.E. (2020). Effect-directed profiling and identification of bioactive metabolites from field, in vitro-grown and acclimatized *Musa* spp. accessions using high-performance thin-layer chromatography-mass spectrometry. J. Chromatogr. A.

[15] Goplan N.N.V.K., Tan S. (2021). Antioxidant activities, total phenolic content and colour parameters in the aqueous extracts of avocado, banana and papaya leaves. J. Sains Kesihat. Malays..

[16] Handayani R., Fans K., Mastuti T. (2021). Comparison study of antioxidant activity from three banana leaves extracts. J. Teknol. Dan Ind. Pangan.

[17] Oresanya I.O., Sonibare M.A., Gueye B., Balogun F.O., Adebayo S., Ashafa A.O.T., Morlock G. (2020). Isolation of flavonoids from *Musa acuminata* Colla (Simili radjah, ABB) and the in vitro inhibitory effects of its leaf and fruit fractions on free radicals, acetylcholinesterase, 15-lipoxygenase, and carbohydrate hydrolyzing enzymes. J. Food Biochem..

[18] Wu Y.H., Zhuo Z.H., Wang C.X., Liu B.Y., Xu D.P. (2025). HPLC fingerprint analysis of *Zanthoxyllum bungeanum* Maxim. J. Food Compos. Anal..

[19] Qiu J., Li J., Shang S.Y., Zhou P., Leng J. (2025). HPLC fingerprint combined with chemometrics and multicomponent content determination for quality evaluation and control of Huangma Tincture. Phytochem. Anal..

[20] Qian C.J.L., Wang S.Z., Chen H.Y. (2025). Evaluation study of congelex laxative granules based on HPLC fingerprint, multi-component content determination, and chemometrics. J. Pharm. Biomed. Anal..

[21] Zeng M.G., Zheng W.W., Zhang K., Zheng W.Q. (2025). Quality evaluation of Sojae Semen Praeparatum from different origins based on fingerprint and multi-index quantification combined with entropy weight-TOPSIS model. Chin. J. Mod. Appl. Pharm..

[22] Lai C.D., Xia H.L., Zhang Y.H., He Y.T., Wu X.Y., Ye B.C., Yang H., Zhang B. (2026). A comprehensive review on the pharmacological activities and biosynthetic strategies of protocatechuic acid. Life.

[23] Zhang S.J., Gai Z.B., Gui T., Chen J.L., Chen Q.F., Li Y.L. (2021). Antioxidant effects of protocatechuic acid and protocatechuic aldehyde: Old wine in a new bottle. Evid. Based Complement. Altern. Med..

[24] Chaudhary J., Jain A., Manuja R.A. (2013). Comprehensive review on biological activities of *p*-hydroxy benzoic acid and its derivatives. Int. J. Pharm. Sci. Rev. Res..

[25] Li W., Wang X., Yang L. (2022). Esculetin: A review of its pharmacology and pharmacokinetics. Phytomedicine.

[26] Guan X., Mao J., Tang Y., Wang J., Sun R. (2018). Research progress on pharmacological effects of p-coumaric acid. Chin. Tradit. Herb. Drugs.

[27] Gullón B., Lú-Chau T.A., Moreira M.T., Gullón P. (2017). Rutin: A review on extraction, identification and purification methods, biological activities and approaches to enhance its bioavailability. Trends Food Sci. Technol..

[28] Wang Q., Wei H.C., Zhou S.J., Li Y., Zheng T.T., Zhou C.Z., Wan X.H. (2022). Hyperoside: A review on its sources, biological activities, and molecular mechanisms. Phytother. Res..

[29] Wang X., Liu Y., Zhang T., Wu D. (2021). Research progress on chemical constituents and pharmacological effects of Thesium chinense. China J. Chin. Mater. Medica.

[30] Valentová K., Vrba J., Bancírová M., Ulrichová J., Křen V. (2014). Isoquercitrin: Pharmacology, toxicology, and metabolism. Food Chem. Toxicol..

[31] Liu T.X., Li J., Jiang G.J., Dong X.M., Zhu Z.M., Huang G.H. (2017). Regulation of isoquercitrin on inflammatory factors in LPS-induced RAW264.7 cells. Her. Med..

[32] Li H., Zhang Y.H., Tuo B.X., Xu J. (2025). Effects and mechanism of isoquercitrin on vascular remodeling in AngII-induced hypertensive mice. Prog. Biotechnol..

[33] Li H.Y., Pan X., Wang M.C., Li W.J., He P., Huang S., He F.Y. (2024). A novel method for integrating chromatographic fingerprint analytical units of Chinese materia medica: The matching frequency statistical moment method. Digit. Chin. Med..

[34] Zhu Z.F., Fan Q.M., Liu Y.Z., Meng L., Xiao M.F., Zhou Y.Q., Zhou J., He F.Y. (2020). Study on optimization of UPLC fingerprint of classic prescription of Shentong Zhuyu Decoction based on principle of information entropy maximization. Chin. Herb. Med..

