# Supplementary material for: Application of HPLC Fingerprint Coupled with Chemical Pattern Recognition and Multicomponent Quantification for Quality Evaluation of Ethyl Acetate Extract of Banana Leaves
Source: Molecules. 2026 Aug 28;31(17):3023. doi: 10.3390/molecules31173023 (PMC13567616; doi:10.3390/molecules31173023)
Supplement: Supplementary file 1 [file molecules-31-03023-s001.zip › molecules-4450768-supplementary.pdf]

Application of HPLC Fingerprint Coupled with Chemical Pattern Recognition and Multicomponent Quantification for Quality Evaluation of Ethyl Acetate Extract of Banana Leaves

Wen Liu [1,2,3], Liyuan Huang [1], Meifen Ban [1], Fan Liu [1], Ze'en Xiao [1,*] and Xiaotao Hou [1,4,*]

[1] Faculty of Pharmacy, Guangxi University of Chinese Medicine, Nanning 530200, China; wen_l0639@126.com (W.L.); huang6882024@163.com (L.H.); 13481086337@163.com (M.B.); liufan775116747@gmail.com (F.L.)

[2] Key Laboratory of TCM Extraction and Purification and Quality Analysis, Guangxi University of Chinese Medicine, Education Department of Guangxi Zhuang Autonomous Region, Nanning 530200, China

[3] National Demonstration Center for Experimental Traditional Chinese Pharmacology, Guangxi University of Chinese Medicine, Nanning 530200, China

[4] University Engineering Research Center of Reutilization of Traditional Chinese Medicine Resources, Guangxi University of Chinese Medicine, Nanning 530200, China

* Correspondence: xiaoze@gxtcmu.edu.cn (Z.X.); xthou@126.com (X.H.)

Supporting Information

CONTENTS

Table S1. The results of peak area precision test of each component (n=6) 1

Table S2. The results of retention time precision test of each component (n=6) 1

Table S3. Repeatability test results (n=6) 1

Table S4. The results of peak area stability test of each component (n=7) 2

Table S5. The results of retention time stability test of each component (n=7) 2

Table S6. Sample recovery test results (n=6) 3

Table S7. The gradient elution conditions. 4

Figure S1. Curves of information entropy under different detection wavelengths 4

Figure S2. Chromatogram of S4 with acetonitrile–0.1% phosphoric acid as mobile phase (226 nm) 5

Figure S3. Chromatogram of S4 with acetonitrile–0.1% formic acid as mobile phase (226 nm) 5

Figure S4. Chromatogram of S4 with acetonitrile–0.1% acetic acid as mobile phase (226 nm) 5

Figure S5. Chromatogram of S4 with acetonitrile–water as mobile phase (226 nm) 6

Figure S6. Chromatogram of S4 at column temperature of 25 °C 6

Figure S7. Chromatogram of S4 at column temperature of 30 °C 6

Figure S8. Chromatogram of S4 at column temperature of 35 °C 7

Figure S9. Chromatogram of S4 detected at 272 nm (red) and 264 nm (blue) 7

Table S1. The results of peak area precision test of each component (n=6)

No	PCA	PHBA	EC	CA	RT	HR	KGR	IQ
1	673.70	1361.9	1422.9	1330.4	1694.6	2540.2	914.30	2683.9
2	671.80	1364.2	1430.9	1345.0	1706.5	2549.5	919.10	2692.9
3	668.90	1355.0	1422.8	1332.9	1681.1	2520.1	914.00	2643.3
4	670.20	1356.5	1425.4	1333.9	1682.3	2523.5	913.30	2646.0
5	670.70	1358.4	1431.8	1337.6	1692.5	2527.9	918.20	2656.0
6	674.80	1368.4	1431.3	1336.0	1688.2	2532.5	918.70	2654.7
Average	671.70	1360.7	1427.5	1336.0	1690.9	2532.3	926.30	2662.8
RSD%	0.33	0.37	0.30	0.38	0.55	0.43	0.29	0.77

Table S2. The results of retention time precision test of each component (n=6)

No	PCA	PHBA	EC	CA	RT	HR	KGR	IQ
1	11.978	18.135	21.836	31.677	40.702	41.517	42.286	43.113
2	11.895	18.036	21.728	31.556	40.554	41.380	42.134	42.958
3	11.843	17.959	21.693	31.546	40.863	41.643	42.451	43.264
4	11.890	18.015	21.786	31.646	40.930	41.725	42.539	43.356
5	11.874	18.003	21.744	31.561	40.733	41.541	42.334	43.162
6	11.888	18.014	21.735	31.592	40.808	41.619	42.422	43.242
Average	11.895	18.027	21.754	31.596	40.765	41.571	42.361	43.183
RSD%	0.38	0.33	0.23	0.17	0.33	0.29	0.34	0.32

Table S3. Repeatability test results (n=6)

No	PCA (μg/mg)	PHBA (μg/mg)	EC (μg/mg)	CA (μg/mg)	RT (μg/mg)	HR (μg/mg)	KGR (μg/mg)	IQ (μg/mg)
1	8.56	14.62	73.12	25.66	45.50	53.27	23.60	55.26
2	8.61	14.73	73.91	25.89	46.08	53.76	23.71	55.96
3	8.77	15.01	75.29	26.60	47.30	55.48	24.60	57.13
4	8.72	14.87	74.66	26.34	46.79	54.42	24.46	57.25
5	8.77	15.38	75.93	26.55	48.02	56.02	24.85	58.41
6	8.82	15.10	74.50	26.93	47.13	56.00	23.98	57.47
Average	8.71	14.95	74.57	26.33	46.80	54.83	24.20	56.91
RSD%	1.2	1.8	1.3	1.8	1.9	2.1	2.1	1.9

Table S4. The results of peak area stability test of each component (n=7)

No	PCA	PHBA	EC	CA	RT	HR	KGR	IQ
0h	583.20	1418.6	1528.2	1181.8	1491.3	2610.3	1033.9	2728.0
2h	586.70	1426.7	1511.3	1132.1	1492.8	2604.6	1026.8	2723.1
4h	587.20	1420.9	1522.5	1127.9	1480.2	2611.0	1005.7	2712.1
8h	599.30	1434.3	1541.8	1140.6	1496.3	2647.3	1050.0	2796.1
10h	592.60	1429.9	1527.7	1132.9	1485.4	2643.1	1005.3	2719.6
12h	597.10	1436.7	1531.3	1123.3	1488.6	2616.8	999.1	2704.2
24h	592.00	1431.2	1534.7	1134.4	1494.3	2639.6	1015.9	2746.6
Average	591.20	1428.3	1528.2	1139.0	1489.8	2624.7	1019.5	2732.8
RSD%	0.98	0.47	0.63	1.7	0.37	0.68	1.8	1.1

Table S5. The results of retention time stability test of each component (n=7)

No	PCA	PHBA	EC	CA	RT	HR	KGR	IQ
0h	11.787	17.861	21.890	31.374	40.420	41.238	42.084	42.903
2h	11.810	17.861	21.825	31.387	40.450	41.231	42.052	42.859
4h	11.729	17.806	21.760	31.327	40.603	41.371	42.212	43.004
8h	11.775	17.852	21.789	31.286	40.436	41.198	41.986	42.798
10h	11.805	17.902	21.890	31.538	40.946	41.718	42.552	43.359
12h	11.872	17.998	21.960	31.657	40.989	41.796	42.581	43.425
24h	11.766	17.840	21.798	31.399	40.641	41.417	42.250	43.048
Average	11.792	17.874	21.845	31.424	40.641	41.424	42.245	43.057
RSD%	0.38	0.34	0.33	0.41	0.59	0.58	0.56	0.57

Table S6. Sample recovery test results (n=6)

Component	No	Original quantity (μg)	Adding quantity (μg)	Measured amount (μg)	Recovery rate (%)	Average recoveries (%)	RSD (%)
PCA	1	4.354	4.405	8.841	101.9	102.2	0.32
	2	4.354	4.405	8.851	102.1		
	3	4.354	4.405	8.868	102.5		
	4	4.354	4.405	8.865	102.4		
	5	4.354	4.405	8.844	101.9		
	6	4.354	4.405	8.878	102.7		
PHBA	1	7.477	6.665	14.34	102.9	103.6	0.37
	2	7.477	6.665	14.39	103.8		
	3	7.477	6.665	14.39	103.8		
	4	7.477	6.665	14.40	103.9		
	5	7.477	6.665	14.40	103.9		
	6	7.477	6.665	14.37	103.4		
EC	1	37.28	37.86	76.02	102.3	103.7	0.84
	2	37.28	37.86	76.69	104.1		
	3	37.28	37.86	76.60	103.9		
	4	37.28	37.86	76.84	104.5		
	5	37.28	37.86	76.33	103.1		
	6	37.28	37.86	76.87	104.6		
CA	1	13.16	12.71	26.19	102.5	102.0	0.45
	2	13.16	12.71	26.10	101.8		
	3	13.16	12.71	26.20	102.6		
	4	13.16	12.71	26.08	101.7		
	5	13.16	12.71	26.06	101.5		
	6	13.16	12.71	26.08	101.7		
RT	1	23.40	23.84	47.34	100.4	101.6	0.66
	2	23.40	23.84	47.73	102.1		
	3	23.40	23.84	47.80	102.4		
	4	23.40	23.84	47.66	101.8		
	5	23.40	23.84	47.62	101.6		
	6	23.40	23.84	47.56	101.4		
HR	1	27.41	29.20	57.88	104.3	104.3	0.52
	2	27.41	29.20	57.99	104.7		
	3	27.41	29.20	57.91	104.5		
	4	27.41	29.20	57.92	104.5		
	5	27.41	29.20	57.55	103.2		
	6	27.41	29.20	57.93	104.5		
KGR	1	12.10	12.65	24.85	100.8	101.9	0.60
	2	12.10	12.65	25.01	102.1		
	3	12.10	12.65	25.07	102.6		
	4	12.10	12.65	25.02	102.2		
	5	12.10	12.65	25.02	102.2		
	6	12.10	12.65	24.97	101.8		
IQ	1	28.46	30.00	59.60	103.8	103.9	0.58
	2	28.46	30.00	59.79	104.5		
	3	28.46	30.00	59.81	104.5		
	4	28.46	30.00	59.50	103.5		
	5	28.46	30.00	59.34	103.0		
	6	28.46	30.00	59.69	104.1		

Table S7. The gradient elution conditions.

Time（t/min）	Acetonitrile（%）	0.1% Phosphoric acid aqueous solution（%）
0	5	95
24	14	86
42	17	83
47	20	80
65	31	69
85	47	53
100	67	33
110	90	10
115	90	10

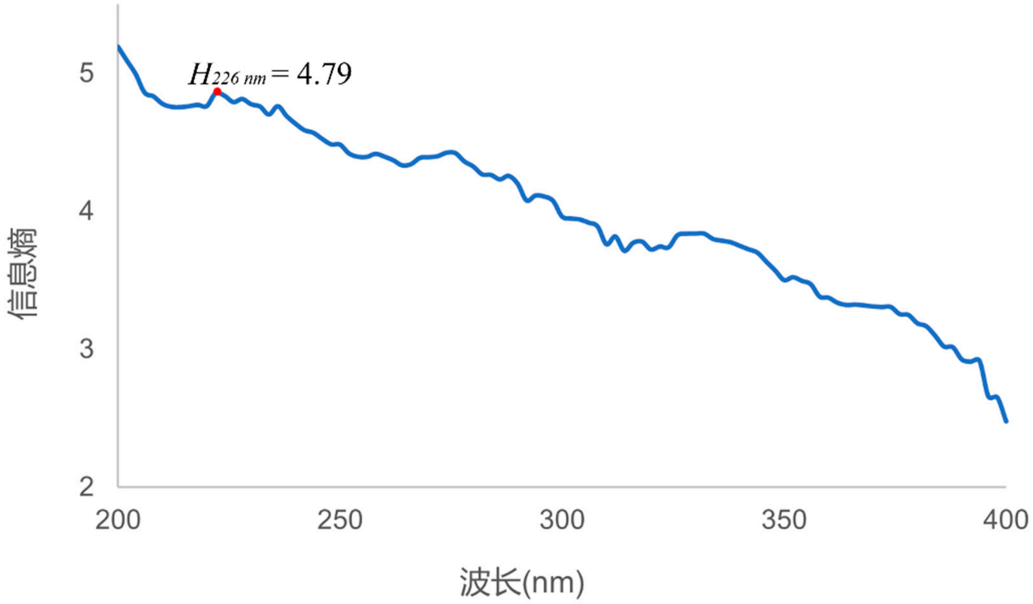

Figure S1. Curves of information entropy under different detection wavelengths

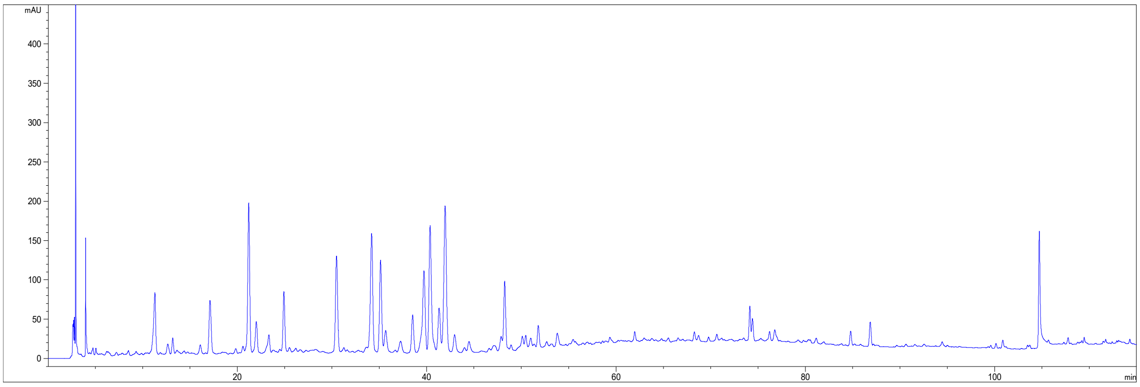

Figure S2. Chromatogram of S4 with acetonitrile–0.1% phosphoric acid as mobile phase (226 nm)

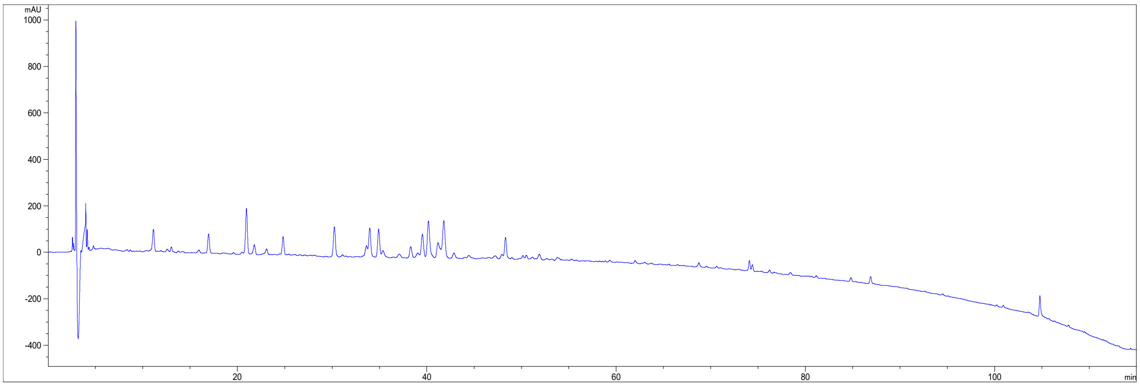

Figure S3. Chromatogram of S4 with acetonitrile–0.1% formic acid as mobile phase (226 nm)

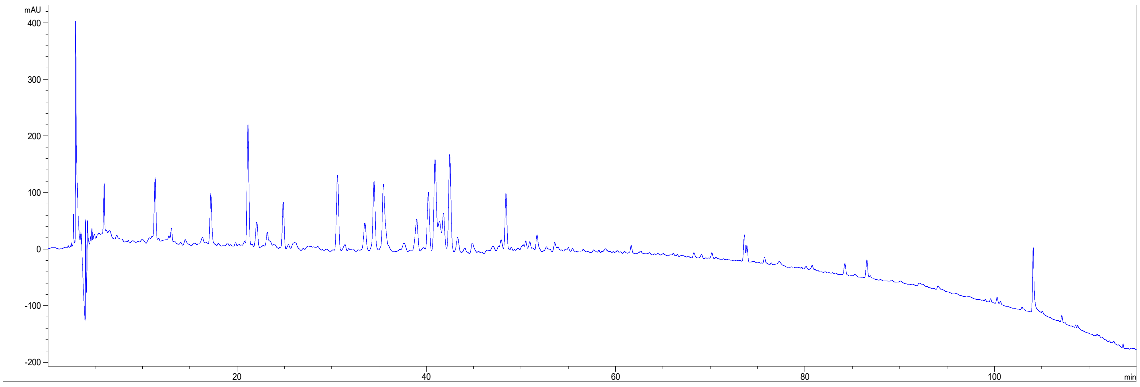

Figure S4. Chromatogram of S4 with acetonitrile–0.1% acetic acid as mobile phase (226 nm)

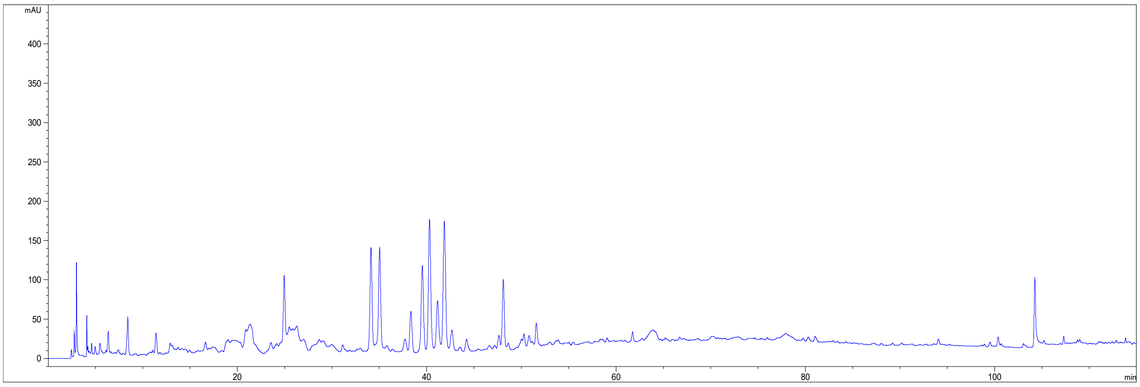

Figure S5. Chromatogram of S4 with acetonitrile–water as mobile phase (226 nm)

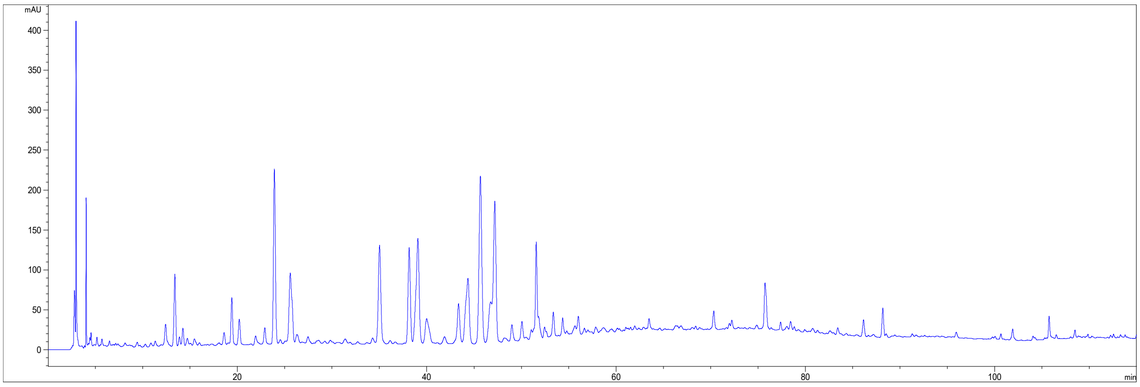

Figure S6. Chromatogram of S4 at column temperature of 25 °C

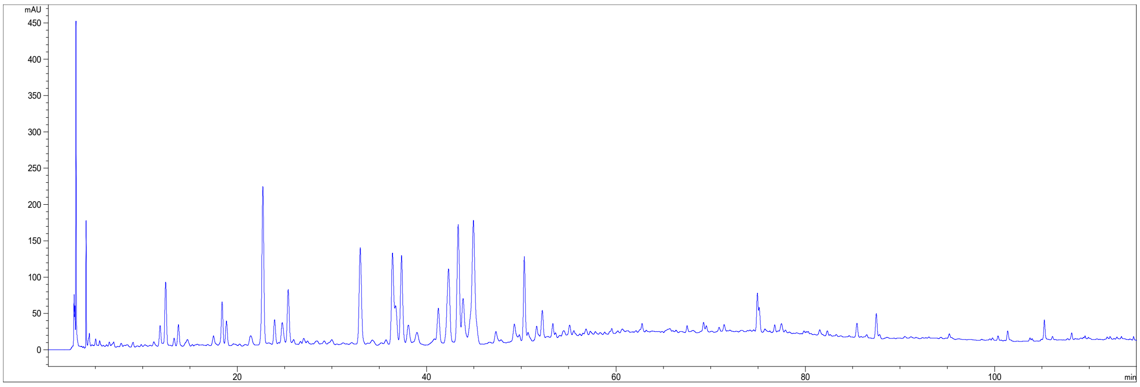

Figure S7. Chromatogram of S4 at column temperature of 30 °C

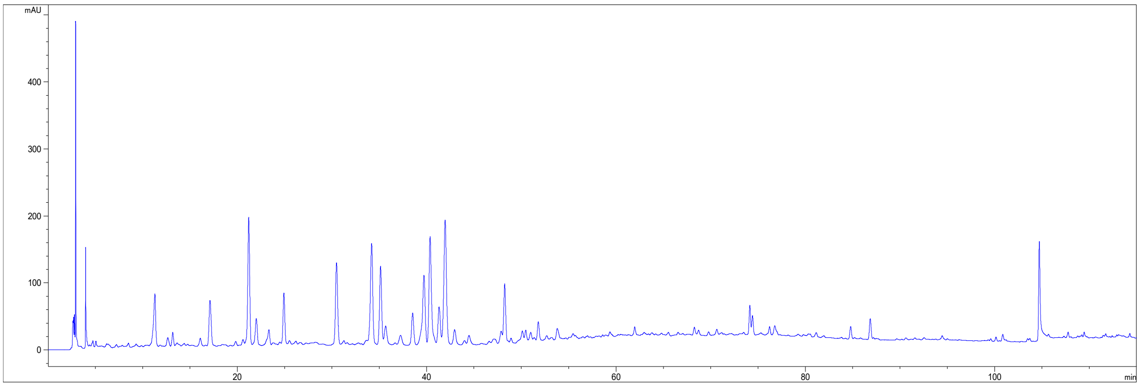

Figure S8. Chromatogram of S4 at column temperature of 35 ℃

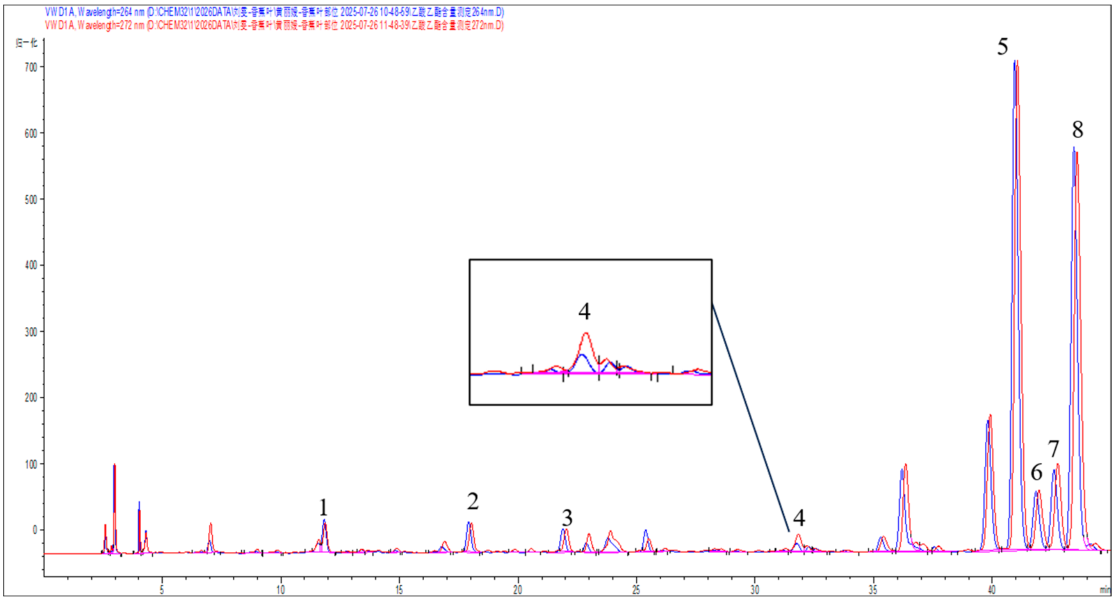

Notes: 1. protocatechuic acid; 2. p-hydroxybenzoic acid; 3. esculetin; 4. p-coumaric acid; 5. rutin; 6. kaempferol-3-*O*-glucorhamnoside; 7. hyperoside; 8. isoquercitrin.

Figure S9. Chromatogram of S4 detected at 272 nm (red) and 264 nm (blue)
